# Report on the Progress of Physiology and Anatomy

**Published:** 1858-01

**Authors:** S. Weir Mitchell

**Affiliations:** Lecturer upon Physiology in the Philadelphia Association for Medical Instruction; Member of the College of Physicians of Philadelphia, etc.


### REPORT ON THE PROGRESS OF PHYSIOLOGY AND ANATOMY.

By S. WEIR MITCHELL, M.D. Lecturer upon Physiology in the Philadelphia Association for Medical Instruction; Member of the College of Physicians of Philadelphia, etc.

IT is proposed in future to present, once a year, to the readers of this journal, a complete analytic review of the general progress of Physiology and Anatomy. At the same time, and as an essential part of this task, we shall endeavor to point out with care whatever advances in these sciences are due to the annually increasing labors of our own countrymen. As a considerable share of the recent European additions to physiology has already been presented during the past year to our readers, we have decided on the present occasion to replace the report, thus rendered for the most part unnecessary, by a statement of the progress of physiology and anatomy in this country. Such a review as we contemplate ought not to lack of interest as a medical and literary record, while, at the same time, we indulge the additional hope that it may be of some use to the profession to ascertain, in this direction, how much or how little we, as a nation, have done for the world of science. With this view, we have analyzed and catalogued every physiological and anatomical essay which has appeared in the journals during the last ten years. We have also selected the best of the papers whose publication occurred previous to 1847, and have sought to arrange them in such a manner as to give to the list some value for purposes of future reference. It has been found quite impossible to collect the titles of the many pamphlets, etc., which have appeared from time to time. In most cases, this omission is of no moment, since such pamphlets are usually mere collections of essays originally made public in the journals. Even with this general limitation to the journal literature of our subject, the task has been no light one, and it is very possible, as the time allotted to us for its completion was but two months, that some essays may have escaped our notice. It is hoped, however, that our list is sufficiently complete to be useful, and that, as a record of how little has been done in past years, and how much more has been done within a recent period, we may find in it a lesson, and the promise of a more hopeful future.

While thus admitting a sense of disappointment at the general result of our inquiry, we have no wish to teach that our countrymen have done nothing for physiology and anatomy. Here and there, in the files of journals through which we have looked, are to be found essays of the utmost value, and often of an excellence quite unapproached in the special subjects of which they treat. Some of these papers are models of the kinds of research which they exemplify, and are to this day of standard authority. It will also be observed, as we progress, that some important physiological subjects have been with us altogether neglected, while others have received an unusual share of attention, either because they involved the interests of races, or because some special circumstances enabled the experimenter to resort to means not elsewhere available. The first of these causes has given us a series of papers upon the anatomical and physiological pecularities of the African, as well as his mortality, fecundity, etc.—questions in which we are largely interested as a nation, and which have obtained illustration, directly or indirectly, from the pens of Morton, Bachman, Cartwright, Dowler, etc. Special advantages of position, and indeed the very necessities of early burial during epidemics in warm climates, have given to Dr. Dowler the opportunity of numerous well-known researches upon capillary forces, post-mortem heat, and post-mortem muscular contractions, etc.; we have thence acquired many facts which might have been long in coming to light had it not been for the New Orleans custom of making post-mortem examinations almost as soon as death has seized upon the frame. On the other hand, no one can read our journals without being struck with the number of theorizing papers, parading long lists of facts derived from the pages of other writers, without an effort to add one new, or freshly invented experimental illustration.

This eager love of appearing in print at all hazards, finds also profuse self-indulgence in dreary controversial papers, which are only too common in some of our journals, and of whose multitudinous dullness no luckless bibliographer has as yet taken the census. Any one who doubts on this point, and who is even remotely open to conviction, is desired to read, in the first place, a lady physiologist, Mrs. Emma Willard, upon "Respiration as *the* Motive Power of the Circulation of the Blood." Few, we fancy, could discover in this good lady's views anything worthy either of refutation or remembrance; but for some reason her supposed discovery excites a buz of mingled admiration and mockery, whose "mortal result" is to be found in eighteen papers, pro and con, which adorn the pages of a northern and a southern journal. The name of the thing in dispute finally comes to be "Hæmatokinety," but the subject remains the same, as any adventurous reader may find to his cost, should he be inclined to this particular method of literary suicide.

Before the year 1856, Dr. Dunglison's well-known text-book was the only complete work upon physiology originally published in this country. It yet remains, after numerous editions, a favorite with the student, and the profession generally, being still remarkable for the fullness and distinctness of its statements, and for the valuable bibliographical references, which, in the form of foot-notes, add greatly to its use as a book of reference.* Within the last year the admirable work of Professor Draper, on Human Physiology, has been given to the professional public. As a whole, this work is worthy of its

* The sale of Dr. Dunglison's medical works is almost unprecedented in the history of medical literature. Several years ago it was stated by Mr. Carey, in an essay on International Copyright, to have reached to 80,000 volumes, and at present it can be stated with greater correctness as extending to at least 95,000 volumes.

author's fame. We might point to some portions, such as the chapter upon the functions of the various parts of the inner ear, as being rather loosely reasoned; yet throughout it is remarkable for originality of view and freshness of expression, and is altogether more interesting as a book than is at all common with scientific text-books.

The older and smaller treatises compiled in this country, such, for example, as those of Oliver and Coates, have long passed out of use, and need claim no further notice. The American works on Human Anatomy, such as those of Wistar, Horner, Morton, and Richardson, are, on the other hand, too well known to require comment, and we shall therefore pass on to the divisions of our subject with but little additional preface.

It will, no doubt, strike the reader as singular, that the two great physiological text-books above named should be here quoted as of American origin. Both the authors, however, have long been residents of this country, although both, like Professor Blake, are of foreign birth. Both of them, also, we believe, consider themselves as ours by adoption, as, without doubt, they are by every other tie of respect and well-merited esteem. Mr. Brown-Séquard has himself claimed the rights of scientific citizenship, if we may so speak, upon the grounds of affection—of intended residence, and of birth. We do not see how we are to throw out such a claim, or, indeed, why we should wish to do so, although his scientific abilities owe their primary training to the land whose language he speaks, and where he so long resided.

With this preface, we propose to review, in turn, each physiological function, and finally to speak of anatomy, briefly indicating what has been done for each upon this side of the ocean, and referring to the principal essays upon the topics of which we are writing.

1. *The Nervous System.*—If, as we have said, and as he himself wishes, we claim Mr. Brown-Séquard as an American writer, the best papers upon the physiology of the nervous system are undoubtedly to be attributed to him. These essays illustrate physiologically the causes of epilepsy, and treat with equal success and originality of the track of sensations in the spinal column. We have not thought it requisite to note the journals in which most of these papers occur, because they have been since partly collected into a small volume in this country, and are set forth most fully in an able report to be found in the *Comptes Rendus* of the Société de Biologie, Paris, 1855. Next to the distinguished physiologist last mentioned, the best-known native writer is, beyond doubt, Dr. Bennett Dowler, of New Orleans. Of his researches upon other functions we shall have frequent occasion to speak as we proceed. His views in regard to the nervous system are so utterly opposed to the commonly received canons of physiology as to make it worth while, at least, to remind the reader of the researches, or rather experiments which placed Dr. Dowler in his present antagonistic position. Dr. Dowler rejects the idea of the centralization in the brain of volition and sensation, and believes in the possibility of volition apart from the brain, as well as in the existence of a diffused sensorium. The experiments upon which these views are based were made chiefly upon alligators, in whom Dr. Dowler believed himself able to recognize the existence of voluntary motion and sensation after the animal employed had been decapitated, pithed, and eviscerated. These experiments have been variously interpreted, according as they who reasoned upon them were or were not believers in the possible existence of combined and associated reflex movements. In either view the experiments are of remarkable interest, and this is in no way lessened by the singular and vivacious mode in which Dr. Dowler relates his results.

Up to the present year we have but few original essays of value to add to our list of researches upon this part of physiology. Among these, however, are Dr. Clay Wallace's essays on the physiology, etc., of the nervous system of the eye. Two papers—the one old, 1838, the other recent—upon the functions of the cerebellum. Dr. M. Michel on the Cerebro-Spinal Fluid, etc. Dr. Campbell's recent essay on the excito-secretory system of nerves, will be considered more fully at the close of our report. Besides these essays, there are also on our index-book some thirty additional papers, which, as a general rule, it is quite unnecessary to name, much less to analyze.

*Brown-Séquard.*—Researches on Epilepsy: Its Artificial Production in Animals, etc.; Boston Med. & Surg. Jour. from Nov. 1856, to Nov. 1857. Republished by D. Clapp, Boston, 1857.

*Brown-Séquard.*—Phys. & Pathol. Researches, 1855, Philadelphia; also Med. Ex., 1852–53.

*Bennett Dowler.*—Nervous Systems, etc.; New Orleans Med. & Surg. Jour., vol. iv. pp. 189–279.—Nervous Action, etc. etc.; New Orleans Med. & Surg. Jour., vol. viii. p. 37.

*N. S. Davis.*—The Cerebellum not Connected with Sexual Instinct; Trans. Am. Med. Assoc., vol. iii. p. 22.

*I. D. Fisher.*—On Cerebellum—Cases of Atrophy of, Coexistent with Sexual Defects; Med. & Phys. Jour., 1838–39, vol. xxiii. p. 352.

*Clay Wallace.*—Nervous System of the Eye; Boston Med. & Surg. Jour., vol. xxxviii. pp. 169, 189, 208, 280.

*M. Michel.*—Cerebro-Spinal Fluid; Charleston Med. Jour. & Review, vol. iii. p. 371.

*H. F. Campbell.*—Excito-Secretory System of Nerves; Prize Essay; Trans. Am. Med. Assoc., vol. x. p. 465, 1857.

*H. F. Campbell.*—The Sympathetic Nerve in Reflex Phenomena; Trans. Am. Med. Assoc., vol. vi. p. 539, 1853.

*John Harrison.*—Sensations and the Relation between Nervous Matter and the Objects of Impressions; New Orleans Med. & Surg. Jour., vol. vi. pp. 425, 697.

*Bennett Dowler.*—Physiology of Subjectivity; New Orleans Med. & Surg. Jour. New Series, vol. viii. p. 64.

*Special Senses.*—Dr. C. Wallace's papers upon the anatomy and physiology of the eye have just been alluded to; his description, etc. of the anatomy and physiological function of the ciliary muscle is still better known, and is to be found in a little volume published in 1852. Dr. Wallace also wrote a small volume on the structure of the eye, with reference to natural theology. Amid seventeen papers upon the special senses, by various authors, we have found but few of interest to any one beyond their writers. Dr. Draper's text-book contains chapters upon the eye and ear, which are remarkable for bold application of recently acquired physical facts to the explanation of ocular and auditory phenomena. We would especially call attention to the chapter upon the eye. Dr. Draper's view of the functions of the inner ear differs greatly from that of Dr. S. Jackson, who has also written thereon with great ingenuity, and who regards the semicircular canals as forming an apparatus for producing interference of the waves of sound, and so preventing the occurrence of confusing echoes in the ear. Dr. Draper assigns this office to the two scalæ of the cochlea, and advocates this view with his usual skill. Professor Alexander contributes to our list an abstract of his researches upon the analogies of touch and sight, which would very well bear extension into a paper of greater completeness. Drs. Loomis, Lovering, Hilgard, Hallinan, Campbell, W. W. Clark, Leaming, and F. Michel have also written upon various points connected chiefly with the sense of sight.

*W. C. Wallace.*—Accommodation of the Eye to Distances, 8vo, 1850.

*W. C. Wallace.*—Contributions to the Physiology and Pathology of the Nervous System of the Eye; Boston Med. & Surg. Jour., vol. xxxviii. pp. 169, 189, 208, 280.

*J. B. Hallinan.*—Adjustment of the Eye to Distances; Boston Med. & Surg. Jour., vol. xlix. p. 324, 1854.

*R. F. Michel.*—Ciliary Processes, Uses of; Am. Jour. Med. Science, 1847, vol. xiv. p. 393.

*Hilgard.*—Contributions to the Physiology of Sight; Trans. Am. Assoc. Adv. of Sci., 1855, p. 251.

*S. Jackson.*—Functions of the Parts of the Inner Ear; Am. Jour. Med. Sci., vol. xxxi. p. 550, 1856.

*E. Loomis.*—On Optical Moving Figures; Trans. Am. Assoc. Adv. Sci., 1851, p. 293. Also on Curious Phenomena of Sight, *Professor Lovering;* Trans. Am. Assoc. Adv. Sci., 1849, p. 369.

*F. Leaming.*—Foramen of Soemmering, Uses of; Am. Jour. Med. Sci., vol. xxx. p. 80.

*Alexander.*—Special Analogies of Sight to Touch; Trans. Am. Assoc. Adv.Sci., 1852, p. 365.

*W. W. Clark.*—Relation between Erect Vision and the Inverted Image on the Retina; New York Jour. Med., 1852, p. 366.

*R. D. Mussey.*—Experiments on a Case of Congenital Absence of the Auditory Meatus in both Ears; Am. Jour. Med. Sci., Feb. 1838, p. 378.

2. *Digestion and Absorption.*—Since an accident, and the fortunate labors of Beaumont conferred physiological immortality upon Alexis St. Martin, we have done almost literally nothing additional in the study of the digestive functions. Excepting, therefore, two very excellent essays by Dr. J. C. Dalton, on certain points in digestion, and two valuable papers by Dr. F. G. Smith, relating experiments recently made by himself upon St. Martin, the journals are barren of original views on this subject. A paper by Dr. J. Jones upon the digestion of albumen, etc., and containing accounts of examinations of the stomachs of many animals, should, however, be also mentioned, since it contains observations upon the contents of the stomachs of animals, of a kind which few physiologists are so situated as to be able to make. This essay has since been incorporated into Dr. J. Jones's "Smithsonian Contributions to Useful Knowledge." Of one or two other essays which belong to the past year, we shall presently speak more at length.

Our medical literature contains but little that is novel upon the subject of absorption. The older essays upon the phenomena of osmotic currents in fluids and gases, are by far the best, and have long since been recognized as authority in the matters of which they treat. More recently, Drs. G. W. Jones and J. Jones have also furnished each a paper upon the osmosis of saline solutions. Drs. Pennock and Blake appear upon our list as the authors of papers upon the absorption of poisons; and among the yet older essays are the reports of Drs. Lawrence, Coates, and Harlan, upon the subject of venous and lymphatic absorption. As far back as the year 1822, we have found a very excellent paper by Dr. Milnor, upon nutritive enemata. It contains many good experiments, and observations long since forgotten; and we regret that our space does not allow us to analyze its contents more completely.

*William Beaumont.*—Physiology of Digestion, with Experiments on the Gastric Juice, 12mo.

*J. K. Mitchell.*—On Penetrativeness of Fluids and Gases; Am. Jour. Med. Sci., 1830–31, vol. vii. p. 36; 1833–34, vol. xiii. p. 100.

*Dr. Togno.*—Endosmosis, etc.; Med. & Phys. Jour., 1829, vol. iv. p. 73.

*R. E. Rogers.*—Experiments on the Blood—Endosmosis of Gases, etc.; Am. Jour. Med. Sci., 1836, vol. xviii. p. 277. *Faust.*—Endosmosis & Exosm. of Gases, 1830; Am. Jour. Med. Sci., vol. vii. p. 23.

*Drs. Harlan, Lawrence, and Coates*, 1821.—Report to the Academy of Medicine upon Absorption. Also *Harlan* —Physical Researches; and *Lawrence* and *Coates*, Med. & Phys. Jour., vols. v. and vi. p. 327.

*J. W. Draper.*—On Endosmosis and Exosmosis; Am. Jour. Med. Sci., August, 1836, p. 276; also Nov. 1837, p. 122; May, 1838, p. 23; Aug. 1838, p. 302.

*Dr. Milnor.*—Experiments and Observations upon Medical and Nutritive Enemata; Med. & Phys. Jour., 1821–22, vols. iii. and iv. p. 10.

*Jas. Moultrie.*—Function of the Lymphatics; Am. Jour. Med. Sci., 1827, vol. i. p. 344, New Series.

*G. W. Jones.*—Endosmosis and Exosmosis as connected with the Action of Purgatives, 1848; Am. Jour. Med. Sci., vol. xv. p. 308.

*J. Jones.*—Exosmosis, etc. of Saline Solutions of Different Densities.—Action of Salines in Living Animals and through Dead Membranes, etc., 1856; Am. Jour. Med. Sci., vol. xxxi. p. 61.

*J. C. Dalton.*—(Digestion.)—Decomposition of Iodide of Starch by the Animal Fluids; Am. Jour. Med. Sci., 1856, vol. xxxi. p. 326.

*J. C. Dalton.*—Digestion of Starch; Am. Jour. Med. Sci., vol. xxxii. p. 555.

*F. G. Smith.*—Experiments on Digestion, Gastric Juice, etc.; Med. Ex., 1856, vol. xii. p. 385.

*F. G. Smith.*—Experiments, etc. on Digestion of Starch in Stomach; N. Am. Med. Chirur. Rev., 1857, vol. i. p. 598.

*J. Jones.*—Digestion of Albumen and Flesh, and the Comparative Anatomy and Physiology of the Pancreas; Med. Ex., 1856, vol. xii. p. 257.

*Harlan.*—Physical Researches, Phila. 1835

*J. B. Stuart.*—Absorption by the Skin; New York Med. Rep., vols. i. and iii., 1810–11.

*R. D. Mussey.*—Phila. Med. & Phys. Jour., vol. i. p. 288.

*Dr. Rousseau.*—Experimental Dissertation on Absorption; Phila. 1800.

*J. J. Allison.*—On the Lymph Hearts of Animals. (A very excellent paper.) Am. Jour. Med. Sci., 1838, vol. xxii. p. 377.

3. *Circulation.*—Here, as we have observed in our prefatory remarks, the papers upon Mrs. Emma Willard's theory reign supreme as to number. Since, however, the profession is pretty well agreed upon the subject of "the respiration as the motive power of the blood," we shall not venture to deal further with the matter, believing, on the whole, that a lady's "motive" is not to be ungallantly scrutinized. The action of the great central propulsive organ, and the causes of its sounds, was made the subject of original inquiry by Dr. Pennock, whose results are chiefly embodied in his edition of "*Hope on the Heart.*" Dr. Pennock has also contributed a useful paper upon the pulse of the aged, showing that the pulse becomes more rapid in the old. Dr. Pennock's views upon this subject are now generally received, but, at the time of the publication of his paper, the profession was singularly fixed in the belief that the pulse of the old was slower than that of the middle-aged adult. It scarcely comes within our range to speak of essays upon the pulse of disease, but we are unwilling to pass by in silence the papers of Drs. Flint and Hooker, both of whom have incidentally illustrated the physiology of the pulse while studying its pathological phenomena. The experiments of Dr. Dowler upon the capillary circulation in man are among the most interesting of which we shall have to speak in this connection. During the prevalence of epidemic fevers in summer in New Orleans, it appears to be the custom to make the *sectio cadaveris* within a very short time after death has occurred. Taking advantage of this, Dr. Dowler made many experiments upon the capillary circulation while the blood was still fluid. Grateful as we are for these researches, it is difficult to accustom oneself to the idea of *post-mortem* examinations where life is absent but an hour or two, or even less, and where the blood flows and the muscles contract as the knife of the dissector divides them.* Dr. Dowler's essays, as well as the older papers of Dr. Draper, upon nutritive attraction, deal principally with the subject of the motive forces which act upon the blood in the capillaries. Dr. Austin Flint, Jr., has also written upon the question of independent capillary forces as aids to circulation, and Dr. Camman has furnished a brief paper upon the peculiarities of the pulmonary capillary circulation. Finally, in a recent prize essay, Dr. H. Hartshorne has brought together almost every important opinion upon the subject of the pulse, etc., and endeavored to show, by reference to the experiments of former observers, that the muscular coats of the arteries by their own rythmical contractions aid the heart in producing the pulse.

* These essays contain, among other matters, Dr. Dowler's account of the extraordinary *post-mortem* gymnastics of an alligator's dissevered head. Singular as the account may be, and most of our readers will remember the surprise it created, the allegations of the New Orleans Physiologist should command that belief which a positive statement, endorsed by many witnesses, is certainly entitled to.

Among the older essays we have also to note a paper by J. J. Allison, upon the existence of a venous pulse in the veins of many inferior animals. This paper is well illustrated, and is in every particular worthy of notice.

*J. W. Draper.*—Chemical Organization of Plants: A Treatise on the Forces which Produce the Chemical Organization of Plants, 4to.

The papers for and against the "Theory" of Mrs. Willard are to be found, by any who desire to read them, in the Boston Med. & Surg. Jour. and the New Orleans Med. & Surg. Jour. for the years 1851–54.

*A. Flint, Jr.*—Capillary Circulation—Phenomena of; Am. Jour. Med. Sci., 1857, vol. xxxiv. p. 13.

*H. Hartshorne.*—The Arterial Circulation: Its Physiology, etc.; Prize Essay of the Am. Med. Assoc., 1856.

*B. Dowler.*—Capillary Circulation, Experimental and Critical Researches upon; New Orleans Med. & Surg. Jour., 1849, vol. v. p. 446.

*J. L. Riddell.*—Observations upon the Capillary Circulation, Cilia, etc. etc.; New Orleans Med. & Surg. Jour., 1852, vol. ix. p. 173.

*J. K. Mitchell.*—Observations on the Pulsations of the Heart of the Sturgeon after death; Am. Jour. Med. Sci., vol. vii. p. 58.

*R. Randolph.*—Valve of Eustachius, Uses of; Am. Med. Jour. Med. Sci., 1851, vol. xxii. p. 96.

*H. A. Ramsay.*—Pulse of Negro Child, etc.; Boston, Med. & Surg. Jour., 1853, vol. xlviii. p. 396.

*S. Weir Mitchell.*—Relations of the Pulse to Conditions of Extreme Inspiration and Expiration; Am. Jour. Med. Sci., 1854, vol. xxvii. p. 387.

*J. J. Allison.*—A Venous Pulse Independent of the Heart and Nervous System; Am. Jour. Med. Sci., 1838–39, vol. xxiii. p. 306.

*C. W. Pennock.*—The Pulse of the Aged; Am. Jour. Med. Sci., July, 1847, vols. xiv. and xv. p. 68.

*T. Robinson.*—Experiments on the Heart of a Monstrous Fœtus; Am. Jour. Med. Sci., Feb. 1832–33, vol. ii. p. 346, New Series.

*J. Wiltbank.*—On the Heart-pulse after Death; Phila. Jour. of the Med. & Phys. Sci., 1824, vol. ix. p. 361.

*C. W. Pennock,* and *R. Moore.*—On the Heart's Action; Med. Ex., Nov. 2, 1839; and also Am. Med. Intel., Dec. 16, 1839.

*Blake.*—Mode of Estimating the Amount of the Blood; Med. Ex., 1849, p. 459.

*John Harrison.*—Review of Opinions upon the Causes of the Coagulation of the Blood, etc.; New Orleans Med. & Surg. Jour., 1847, vol. iv. p. 34.

*Dr. Cammann.*—Peculiarities of the Pulmonary Capillary Circulation; New York Jour. Med., Jan. 1848.

4. *Secretion, Respiration, etc.*—We have indicated below the titles of the only good papers upon these functions with which we are furnished by the journals of the last ten years. To Dr. Dalton we owe a short but clear essay upon the movements of the glottis during respiration. He believes its contraction and expansion to occur synchronously with the expiratory and inspiratory acts, and thinks that the death which follows section of the pneumogastrics is partly owing to a paralysis and consequent collapse of the lips of the glottic opening. We ought also to refer to a brief paper by Dr. Draper, upon the action of the bronchial muscular fibres, as aiding in the diffusion of gases through the lungs.

We have already spoken of the few investigations of the gastric juice which we possess, and, we regret to add, that the physiology of the secretions generally has received but little attention on this side of the water. The report upon the lacteal secretion, made to the American Medical Association, is miserably meagre, and adds but little to our stock of information upon this important subject. Dr. Dalton's recent paper upon the bile we shall consider in our report upon the physiology of the past year. The secretion of the kidneys seems to have attracted a larger share of attention. The best American papers upon its ingredients and their relative proportions are of recent origin, scarcely one having been written before the year 1847. The well-known essays of Dr. Kane upon Kiesteine certainly antedated this period, but, with this exception, we have nothing to point to until we reach the contributions of Drs. Fricke, Hammond, and Draper. All of these gentlemen have experimented upon the relation of urea and uric acid to the retrograde metamorphosis of muscles, and, unfortunately, their results show the most singular diversity. Dr. Draper's paper is very well and carefully arranged, while the final experiments in Dr. Hammond's first essay are the most ingenious attempts to clear up the difficult matter they illustrate, with which we are acquainted. Dr. Hammond has further experimented upon the excretion of the phosphates, and the effects of tea, coffee, and intellectual labor upon the composition of the urine. Dr. J. Jones, of Savannah, whose name we have had frequent occasion to recall, has also furnished us with a series of analyses of the urine of animals, as well as with some very valuable tables of the relative weight of the kidneys and other organs, as compared with that of the whole body of the animal. As usual, we have referred below to other papers of lesser note, and we shall again have occasion to allude to a recent essay of Dr. Isaacs, on the Anatomy of the Renal Organs, as the best paper of this description which our medical literature has yet furnished.

*E. K. Kane.*—On Kiesteine; Am. Jour. Med. Sci., July, 1842, p. 37.

*McPheeters* and *Perry.*—On Kiesteine; Am. Med. Intel., March, 1841, p. 369. Also *S. T. Elliott*, in New York Med. Jour., etc., vol. i., Third Series.

*J. C. Dalton.*—On the Constitution and Physiology of the Bile; Am. Jour. Med. Sci., 1857, vol. xxxiv. p. 305.

*J. C. Dalton.*—Movements of Glottis in Respiration, 1854; Am. Jour. Med. Sci., vol. xxviii. p. 75.

*J. Jones.*—The Kidney and its Excretions, etc., 1855; Am. Jour. Med. Sci., vol. xxix. p. 295.

*J. W. Draper.*—On Respiration—Function of the Bronchial Muscular Fibres, 1852; Am. Jour. Med. Sci., vol. xxiii. p. 314.

*N. S. Davis.*—Secretion of Milk in Health and Disease, etc. etc.; Trans. Am. Med. Assoc., 1855, vol. viii. p. 535; also 1856, vol. ix. p. 415.

*W. A. Hammond.*—Relations between the Amounts of Urea and Uric Acid, 1855; Am. Jour. Med. Sci., vol. xxix. p. 119. Also Urological Contributions, 1856; Am. Jour. Med. Sci., vol. xxxi. p. 330.

*W. A. Hammond.*—Excretion of Phosphoric Acid by the Kidneys; N. A. Med.-Chir. Review, vol. i. p. 730.

*G. T. Elliott.*—Urine in Fœtal Life, 1857; Am. Jour. Med. Sci., vol. xxxiii. p. 555.

*J. C. Draper.*—Is Muscular Motion the Cause of the Production of Urea? 1856; New York Jour. Med., vol. xvi. p. 147, New Series.

*C. E. Isaacs.*—Structure and Physiology of the Kidney; Trans. New York Acad. Med., 1856, vol. i. p. 377; Also, On Functions of the Malpighian Bodies of the Kidney, Feb. 4, 1857, p. 437.

*Booth* and *Boyé.*—Action of Benzoic Acid on the Urine, etc.—Does not decrease the Uric Acid; Trans. Am. Phil. Soc., vol. ix. part ii., 1845.

5. *Nutrition, Growth, Metamorphosis, etc.*—The physiological history of the blood has received some attention at the hands of Dr. J. Jones, in the essay so frequently alluded to, and we are also indebted to various authors for observations upon its microscopical appearances.

In connection with the development, growth, and metamorphosis of tissues, we have but little to point to until the recent appearance of several papers by Hammond, and one by the younger Draper, to which we have already alluded. Previously to these, we have four papers upon cell-life, and allied subjects, by the late Dr. W. J. Burnett; they possess more value as reviewing and enforcing the views of others than from any marked or distinctive originality. Many observations on development, on the blood, on ova, and on other kindred matters, have been made by Dr. Riddell, of New Orleans. Dr. Riddell's papers are, however, usually composed of isolated observations, and are deficient in classification, and in that multiplied and careful observation of single facts which alone can give to such essays a permanent value. This is the more to be regretted, as we do not doubt that an efficient use of Dr. Riddell's known skill and ingenuity would give to our science some more useful proof of abilities as an observer, which are too well known to be left unemployed.

The essays upon the products of the retrograde metamorphosis of tissue, as just now hinted, have been already mentioned in speaking of the kidneys and their excretion; we shall, therefore, content ourselves with the mention of Dr. Hammond's examination of the physiological effects of tea, coffee, alcohol, and tobacco upon the amount of the several elements into which the decaying tissues are supposed to be resolved. Dr. Hammond's conclusions in regard to tea, coffee, and alcohol accord on the whole with those of the recent German observers. His observations upon the effects of smoking tobacco we believe to be original with him. They lead him to the following conclusions:—"That tobacco, when the food is sufficient to preserve the weight of the body, increases that weight, and when the food is not sufficient, and the body in consequence loses weight, tobacco restrains that loss." The value of such researches are of course dependent wholly on the accuracy of the observer and the efficiency of his precautions against fallacies. It ought certainly to be a comfort to the tobacco-consuming millions to know, that with the virtue of organic instinct they are practicing so curious an economy in tissue. Next in value to the papers just named, and like them, subject to the only true criticism on experiments, namely, their repetition in other hands, are the observations, etc. of Dr. Jones upon the effects of starvation, etc. upon cold and warm blooded animals.

*R. D. Mussey.*—Effects of Alcohol in Health and in Disease; Trans. Am. Med. Assoc., vol. viii. p. 573.
*N. S. Davis.*—Assimilation and Nutrition, 1851; North-West Med. & Surg. Jour., Sept. 1851.
*W. J. Burnett.*—Blood Corpuscle-Holding Cells, and their Relation to the Spleen; Trans. Am. Assoc. Adv. Sci., 1853, p. 224.
*J. L. Riddell.*—Histology of Red Blood; Trans. Am. Assoc. Adv. Sci., 1855, p. 239.
*W. P. Hort.*—On the Independent Vitality of the Blood, 1850; New Orleans Med. & Surg. Jour., vol. vi., p. 582; vol. vii. p. 1; vol. viii. p. 164.
*J. Garrie.*—Electrical Conditions of Arterial and Venous Blood, 1854; New Orleans Med. & Surg. Jour., vol. x. pp. 584, 738.
*J. Jones.*—Physical, Chemical, and Physiological Investigations upon the Vital Phenomena, Structure, and Offices of the Solids and Fluids of Animals; Am. Jour. Med. Sci., vol. xxxii. p. 13; also Smithsonian Contributions, 1856.
*W. J. Burnett.*—The Cell: Its Physiology, etc. etc., as deduced from original observations; Trans. Am. Med. Assoc., vol. vi. p. 645. Prize Essay.
*W. J. Burnett.*—New Points in the Morphology of Cells; Trans. Am. Assoc. Adv. Sci., 1849, p. 261.
*W. J. Burnett.*—Cell Segmentation; Trans. Am. Assoc. Adv. Sci., 1853, p. 232.
*J. L. Riddell.*—Origin of Capillary Vessels, etc. etc.; Trans. Am. Assoc. Adv. Sci., 1853, p. 244; also New Orleans Med. & Surg. Jour., vol. ix. p. 173.
*W. J. Burnett.*—The Development, Nature, and Function of Epithelial Structures; Am. Jour. Med. Sci., vol. xx. p. 70, 1850.
*W. H. Byford.*—Physiology of Exercise; Am. Jour. Med. Sci., vol. xxx. p. 32, 1855.
*W. A. Hammond.*—Effects of Alcohol and Tobacco on Nutrition, etc., 1855; Am. Jour. Med. Sci., vol. xxxii. p. 305; also see two essays on Secretion, etc.
*W. A. Hammond.*—Value and Physiological Effects of Albumen, Starch, and Gum, in Nutrition, etc. etc., 1857; Trans. Am. Med. Assoc., vol. x. p. 513; Prize Essay.
*W. J. Burnett.*—Tissue and its Retrograde Metamorphosis, 1851; Am. Jour. Med. Sci., vol. xxii. p. 22.
*W. S. Bowen.*—The Parent Tissue and the Blastema; N. Am. Med.-Chir. Review, vol. i. p. 719.

6. *Calorification.*—Among the American investigators of this function are several of the ablest of our physiological essayists. Laying aside Dr. Metcalf's book, which, although by an American, was first published, and, we think, written in London, we proceed to the valuable essays of Dr. Dowler, on post-mortem and life-temperatures. This is a series of articles every way worthy of a fuller comment, and of publication in book form. Most of them refer to Dr. Dowler's curious discovery of the rise of temperature after death by yellow fever and cholera, observations which stand alone, if we except two or three cases of an elevated post-mortem temperature, reported by Dr. Davy, and supposed by him to have existed during life, and not to be due, as Dr. Dowler has shown, to changes following soon upon dissolution. Dr. Dowler's habit of adventurously exploring the body almost immediately after death, saved him from this error, and enabled him to prove the gradual rise of the thermometer after life had departed.

The recent researches of Dr. Dowler are far more open to criticism, and it is impossible to read without surprise some of his statements in regard to the lowering effect upon the whole system, of applications of cold to one or more parts of it. We are thus informed that cold air, or cold water even, partially applied, in rooms with or without fire, causes a reduction of the temperature sometimes 33° or 34° below the natural heat,—an amount of depression which we are scarcely inclined to suppose possible, and which, at the least, should not pass by without cautious question.

The last paper to which we shall allude, is at the same time one of the best, and proceeds from the pen of Dr. J. Le Conte. It is an examination of the causes of the action of cold upon plants, and is most carefully and elaborately handled, with ingenious and ample experimental illustrations. Professor Payne, of New York, has written a short paper upon a kindred subject—the Temperature of our Forest Trees in Winter. The researches of M. Brown-Séquard upon Human Temperatures are to be found in his published essays. Here, as elsewhere, we have thought it unnecessary to refer our readers to the many essays through which this bold and able physiologist has cast light upon the obscurities of so many of the phenomena of life.

*B. Dowler.*—Calorification, 1855—Experimental Researches upon, etc.; New Orleans Med. & Surg. Jour., vol. xii. pp. 54, 205, 290, 470, 759, 601.

*E. Stevens.*—Temperature of the Body; Boston Med. & Surg. Jour., vol. xli. p. 140.

*W. H. Ford.*—Physiological Uses of Caloric, 1855; Charleston, Med. Jour. & Rev. vol. x. p. 768.

*J. Le Conte.*—Causes of the Endurance of Cold by Certain Vegetables, etc. etc., 1852; Trans. Am. Assoc. Adv. Sci., 1852, p. 338.

*J. Barnes.*—On Human Temperatures; Am. Med. Intell., 1839, p. 346.

*B. Dowler.*—Calorification—Post-mortem; W. J. Med. & Surg., June and Oct. 1844.

*B. Hensley.*—Post-mortem Temperature; Med. Ex., 1846, p. 149.

7. *Reproduction.*—Here, again, we have a remarkable deficiency in good or useful physiological papers. While the practical points of midwifery have obtained repeated and numerous observers and essayists, the physiology of the organs concerned has been almost neglected—so far, at least, as original research is concerned. One single excellent essay, by J. C. Dalton, upon the Corpus Luteum of Pregnancy, is authority upon the subject of which it treats;* one or two essays on Kiesteine, and a few minor papers in addition, complete the meagre catalogue, and leave us no temptation to linger on so barren a field. We have thought it, however, not improper to add to this list the titles of certain essays upon the effects on offspring of marriages of consanguinity, a subject which has only begun to receive the attention it deserves. A very good paper upon the subject will be remembered by many of our readers as due to the pen of Dr. Bemiss.

*J. C. Dalton.*—Corpus Luteum of Menstruation and Pregnancy; Trans. Am. Med. Assoc., 1851, vol. iv. p. 547; Prize Essay.

*M. Michel.*—On Menstruation, with a Case of a Corpus Luteum in Process of Formation, and Coincident with Menstruation; Charleston Med. Jour. & Rev. vol. iii. p. 21. Also, An Early Ovum Described; Am. Jour., Med. Sci., vol. xiv. p. 330.

---

* Dr. Dalton's essay is well known and often quoted. It is an honest and careful paper, and needs no praise from us.

*C. D. Meigs.*—On the Corpus Luteum, 1847; Am. Jour. Med. Sci., vol. xiii. p. 245.
Reproduction—How Affected by Consanguinity—Marriage of Cousins; Am. Assoc. Adv. Sci. 1855, p. 236.
Consanguinity—Evil Effcts of Marriages of, *S. M. Bemiss*, 1857; North American Med.-Chirur. Rev., vol. i. p. 97.

8. The natural history of man as a whole, and the physiological and anatomical peculiarities of races, is a subject which legitimately belongs to the scope of this essay. The inquiry with us has taken a very natural direction, and has furnished us with many essays upon the peculiarities of the negro race, in health and disease. Unhappily the matter has generally been treated in a vague, unscientific way, and the essays in question are only too often notable for their utter want of multiplied and classified facts. These remarks, of course, fail in application to the well-known works of Dr. Morton, and to the essays of Dr. Nott, in the two works edited by Mr. Gliddon. The reports, etc. of Dr. Cartwright, which bear upon this matter, are of too fanciful a character to hold rank as sober scientific papers, and we are so far without a single full examination of the many external differences which characterize the negro race.* Where, for example, are the records of the comparative weights, vital capacities, heights, etc. of the servile and the dominant race? Certainly we are without full details of such general differences as these, and they who examine curiously the essays, whose titles are given below, will feel how much is yet to be done in that direction, and how far more thorough such attempts must be than any which have preceded them.

*B. Dowler.*—Vital Statistics of Negroes, 1856; New Orleans Med. & Surg. Jour., vol. xiii. p. 164.
*S. A. Cartwright.*—Report on the Diseases of the Negro, and his Physiological Peculiarities, 1851; New Orleans Med. & Surg. Jour., vol. vii. p. 691; vol. viii. pp. 187, 369.
*S. A. Cartwright.*—Negro Peculiarities—Effect of Alcohol on the Negro, etc., 1853; New Orleans Med. & Surg. Jour., vol. x. p. 150; also vol. ix. p. 195.
*Grier.*—The Negro and his Diseases, 1853; New Orleans Med. & Surg. Jour., vol. ix. p. 752.
*B. Dowler.*—Vital Statistics of the Negro, 1856; New Orleans Med. & Surg. Jour., vol. xiii. p. 164.
*J. Neill.*—On Peculiarities of African Crania; Am. Jour. Med. Sci., 1850, vol. xix. p. 78, and Trans. Phil. Col. Phys., 1851, vol. i. p. 80, New Series.
*H. A. Ramsay.*—Cranial Dimensions of Negro Child, 1853; Boston Med. & Surg. Jour., vol. xlviii. p. 396.
*E. M. Pendleton.*—Comparative Fecundity of the Caucasian and African Races, 1851; Charleston Med. Jour. & Rev., vol. vi. p. 351. Also on these and allied subjects

* In one of his papers, Dr. Cartwright arranges the diseases of the negro, and treats of them in turn. He describes with complete gravity one disease of the negro which is found only on the plantations of masters who are either too lenient or too severe. This obscure malady is christened solemnly Drapetomonia, from *δραπέτης*, a runaway slave, and *μονια*, madness, etc.

connected with the African race. See the Essays of *Morton* and *Bachman*, in the Charleston Med. Jour. & Rev. during 1850–51; also *Dr. Nott's* contributions to *Mr. Gliddon's* two works.

*S. H. Dickson.*—Statistics of Weight and Height in the South, 1857; Charleston Med. Jour. & Rev., vol. xii. p. 607. These statistics have reference to the white race alone.

*P. A. Browne.*—Classification of Mankind by the Hair and Wool of their Heads, Philadelphia, 1850.

*D. Wilson.*—Displacement and Extinction among the Primeval Races of Man; Canadian Jour. of Sci. Indust. and Art; Toronto, May, 1856.

*J. Broune.*—The Aborigines of Australia; Canadian Jour. Sci. Indust. and Art, May, 1856.

*D. Wilson.*—Supposed Prevalence of one Cranial Type throughout the American Aborigines; Nov. 1857; Canadian Jour. Sci. Indust. and Art.

*C. P. Johnson.*—Description of Skulls of Flathead Indians from Columbia River; Virginia Med. & Surg. Jour.; Feb. 1854. Also Papers by *B. H. Coates* and *J. Parish,* On the Effects of Confinement on the Negro; Trans. Am. Phil. Soc., 1843, vol. iii. p. 143; 1849, Trans. Am. Med. Assoc., vol. ii. For other references to American essays on ethnological subjects generally, see Dunglison's Physiology —Art. Varieties of Mankind, Seventh Edition.

We had hoped to notice here certain statistical papers which bear upon the subject of the general physiological history of man, but some limitation is necessary, and we have, therefore, been obliged to refrain from enumerating the essays to which we refer. Among the best of them, and remarkable for originality and conciseness, are the well-known papers of Dr. Emerson, upon the Increase of Births after Epidemics, etc.

---

### ANATOMY.

THE human body is now so well known, and has been so often vigilantly dissected, that it is almost as vain to expect discoveries in special anatomy as to look for those of new rivers in long-settled countries. On this account we can only point to three papers descriptive of distinct anatomical novelties of any moment. We refer to Dr. Horner's description of the tensor tarsi; Dr. Brinton, on an undescribed valve in the right spermatic vein,—a discovery of considerable interest, as affording an explanation of the common occurrence of varicocele on the left side; and, finally, an essay by Dr. Godman, on an azygos vein of the back. To these we may add the older papers of Godman on regional anatomy; Dr. Horner's essays upon the odoriferous glands of the axilla, and upon the mucous membrane of the intestines; and a number of papers by Dr. W. I. Burnett, on various histological subjects, already alluded to. Several papers by Dr. Leidy are also enumerated below. They are, in some instances, authoritative upon the subjects of which they treat, and are so well known as to require no further comment at our hands. We refer more especially to the essays upon cartilage, and upon the liver.

Two of the best of our anatomical contributions are the recent essays of Dr. Isaacs, upon the pleura, and upon the histology of the kidney.

We have also to thank Dr. J. Jones for some very industrious and elaborate tables of the relative weights of organs; (*See* Smithsonian Contributions.) It is almost needless to add, in conclusion, that we have said as little as possible in regard to essays upon comparative anatomy and physiology, where these did not directly illustrate corresponding points and functions in the human species.

*J. Leidy.*—Anatomy of Articular Cartilage, 1849; Am. Jour. Med. Sci., vol. xvii. p. 277.

*J. Leidy.*—Comparative Anatomy of the Liver—Measurement of Liver-Cells, etc.; Am. Jour. Med. Sci., 1848, vol. xv. p. 13.

*J. Leidy.*—Peculiar Bodies Observed in the Skin, etc. of the Human Subject, 1851; Am. Jour. Med. Sci., vol. xxi. p. 150.

*J. Leidy.*—Anatomy of the Larynx, 1846; Am. Jour. Med. Sci., vol. xii. p. 141.

*W. J. Burnett.*—Form and Mode of Development of the Kidney in Vertebrata; Trans. Am. Assoc. Adv. Sci., 1853, p. 184. See Nutrition, etc.

*J. Neill.*—Mucous Membrane of Stomach, 1851; Am. Jour. Med. Sci., vol. xxi. p. 13. Also Mucous Membrane of Colon and Appendix Verm. Cœci, 1851; Med. Ex. vol. vii. p. 85.

*J. H. Brinton.*—Description of a Valve at the Termination of the Right Spermatic Vein, etc., with Remarks on its Relation to Varicocele; Am. Jour. Med. Sci., vol. xxxii. p. 111.

*W. R. Handy.*—A New Muscle, 1850; Boston Med. & Surg. Jour. vol. xlii. p. 97.

*G. Buck, Jr.*—The Sheath of the Penis, 1846; Trans. Am. Med. Assoc., vol. i. p. 367.

*Dr. W. E. Horner.*—Tensor Tarsi Muscle; Med. & Phys. Jour., vols. xxiii. and xxiv. p. 70.

*Dr. W. E. Horner.*—Odoriferous Glands of Negro; Am. Jour. Med. Sci., 1846, p. 13.

*C. J. Johnston.*—Contrib. to Minute Anat. of Teeth; Jour. of Dent. Sci., Balt.; July, 1857.

We have endeavored, in this short review, to give some idea of what has been done in this country for physiology and anatomy. In so doing we have analyzed pretty fully all the papers published in journals during the last ten years, and have also indicated, at least, the titles of the principal essays before that time, whenever they were in any way entitled to the distinction. At best, it is a melancholy catalogue; and considering the adventurous ingenuity of the national mind, as well as the solid character of its achievements in other lines of scientific research, it is not easy to see why a science so eminently experimental as physiology, should be able to boast so few active laborers and so small a number of conspicuous results. Still, even the last year shows a remarkable improvement, and we may hope, at least, that he who ten years hence reviews the earnings of our physiological workers, may have a larger sum of original results to cast into the lap of the great Mother Truth.

---

*Extent of the Pleura above the Clavicle.* By C. E. ISAACS, M.D., Demonstrator of Anatomy in the University of the city of New York, etc.

WE have great pleasure in asking the attention of our readers to this very industrious and useful paper. "If," says Dr. Isaacs, "any well-informed physician or surgeon were asked, What is the extent of the pleura above the clavicle? or, What is the height and width of the pleural sac in that situation? he would probably answer, that the pleura generally rises about an inch, or perhaps an inch and a half, above the first rib; that its width here was variable and uncertain, and that he was not even sure that it usually reached above the clavicle." After quoting many authors to prove how generally received is this opinion, Dr. Isaacs describes the mode in which he investigated the point in question.

After recording the necessary measurements in one hundred cases, he proceeded to compare them for the first time, and to draw thence his conclusions.

From these data, thus honestly treated, Dr. Isaacs obtained the following conclusions:—

1. That the top of the pleural cavity does not always present a dome-like shape, but occasionally forms cul-de-sacs, which extend upward, like the fingers of a glove, into the recesses of the neck. Sometimes, also, these are prolonged laterally across the median line *to the left side*, and pass upward, either between the bodies of the cervical vertebræ and the œsophagus, or between the œsophagus and the trachea. The pathological and diagnostic applications of the whole of the information above quoted must present themselves at once to every thoughtful reader.

2. In 100 cases the pleura rose, in 23, two inches, and upward, above the clavicle. Of these, 14 were on the right side, 4 on the left, and 5 on both.

3. In 5 cases only, of the 100, did the pleura fail to rise above the clavicle.

4. In 11 cases the sternal or inner edge of the right pleura extended across, and to the left of the median line.

Dr. Isaacs' observations render it probable that a great height of pleura is to be looked for most commonly in persons with very long necks. The remainder of the paper is taken up with an account of the relations of the third division of the sub-clavian artery to the pleural sac.

The essay thus briefly analyzed is one of the most careful and industrious papers upon an anatomical subject which recent medical literature has furnished. The clearness and brevity of Dr. Isaacs' statements are pleasant enough to meet with, in these days of profuse writing and multiplied theories.

---

1. *Researches into the Structure and Physiology of the Kidney.** By S. C. E. ISAACS, M.D., Demonstrator of Anatomy in the University of the city of New York. Read March 5th, 1856.
2. *On the Function of the Malpighian Bodies of the Kidney.** By the same author. Read February 4th, 1857. Illustrated by forty-seven wood-cuts.

IN the commencement of his treatise, Dr. Isaacs remarks, "That he was induced to enter upon a long-extended investigation of the minute structure of the kidney from the fact that the highest authorities were then, and still are, at variance with regard to some of the most important points in the anatomy of this organ. Thus the connection of the Malpighian body with the uriniferous tube, so strongly maintained by Bowman, is denied in toto by Hüschke, Müller, and Hyrtl of Vienna. The existence of ciliated epithelium in the uriniferous tubes of the higher animals, of nucleated cells upon the Malpighian

* From the Transactions of the New York Academy of Medicine, vol. i. part ix.

coil or tuft, of a fibrous matrix, and the arrangement of the venous plexus, are subjects upon which much difference of opinion still exists among anatomists. It was evidently necessary that these points should be determined before we could arrive at a satisfactory physiology and pathology of the kidney." The cause of this difference of opinion among such excellent observers seemed to be that their means of investigation were insufficient. Thus some relied altogether upon the appearances obtained by injection, others trusted to those derived from specimens, which were viewed as transparent objects under the microscope. After very many experiments upon the tissues of the kidney, with various chemical reagents, Dr. Isaacs was enabled to render minute portions of the organ beautifully transparent; and by combining the use of injections with the above processes, obtained many new and exceedingly clear and satisfactory views of its structure, numerous plates of which are here given. The details of the various plans and processes for this purpose are related in the work, to which, for want of space, we can only refer. After alluding to some well-known points in the anatomy of the kidney, the author next examines the epithelium lining the uriniferous tubes. To this great importance has been given by the highest modern authorities, upon the diseases of the kidney, and especially by Bowman and G. Johnson. According to these writers, the urine tubes are lined by spheroidal, or, as they term it, glandular epithelium. Acccording to the observations of Dr. Isaacs, the epithelium, both in the convoluted and straight tubes, is of the "*pavement* or *tessellated*" variety, and he infers that, when it is met with in the rounded form, "either that the kidney is not fresh, or is in a pathological condition, or that the epithelium has been changed by the mode of examination." It is, however, very difficult to obtain the organs in such perfectly normal condition as to show the epithelium in a satisfactory manner, and accordingly every exertion was made to obtain the healthiest specimens for this purpose; very carefully executed plates of the appearances under the microscope are here given. In frogs, snakes, turtles, etc. etc., the uriniferous tubes are lined by *ciliated* epithelial cells, by the action of which the current of urine in the tube is directed and urged onward to the ureter. The existence of such cells in man and the higher animals is still a much-disputed point among anatomists. Dr. Isaacs states, that after making many examinations in order to determine this question, he "resorted to the large establishments in this city for killing oxen, sheep, dogs, rats, etc., and examined the kidneys *immediately* after the death of the animal." The result was that ciliary motion was occasionally observed in dogs, and sometimes in the kidneys of sheep and oxen, "but that it is in them very imperfect, or, as it might be said, in a rudimentary condition."

Several views are given of the arrangement of the venous plexus, and its relation to the tubes and Malpighian bodies, which we pass over.

"*One of the most difficult questions in the minute anatomy of the kidney is, Whether any connection exists between the Malpighian body and the uriniferous tube? and if so, What is the nature of that connection?*" To determine this point, various processes are given, and the result, or the appearances under the microscope, are delineated in fifteen plates, very carefully drawn from nature. The conclusion is, that the description given by Bowman is correct, viz.

that the uriniferous tube terminates by forming an expanded capsule, which embraces the Malpighian tuft or coil of capillaries. This opinion was confirmed beyond the slightest doubt "by very many and repeated observations conducted almost daily for the last twelve months." These specimens and appearances under the microscope were also witnessed by many of the best microscopists in the city of New York, to whom reference is given in the treatise.

There is still much difference of opinion among anatomists, "whether nucleated cells exist upon the Malpighian tuft or coil." An important point in physiology depends upon the settlement of this question. Dr. Isaacs remarks that he is not aware that any anatomist has satisfactorily demonstrated epithelium upon this tuft, in the higher animals. To determine this the following processes were adopted:—1. "By injecting watery and etherial solutions into the ureter, I succeeded in bursting the capsule, the Malpighian tuft or coil having been previously only slightly, and as was intended, imperfectly injected from the artery. Epithelial cells could then be seen upon the uninjected and transparent edges of the tuft." This was in the black bear. 2. By tearing off the capsule with a needle, small nucleated cells could be seen on the reversed and internal surface of the capsule, while the tuft remained covered by cells of larger size than those on the interior of the capsule. The cells of the tuft and those of the capsule were differently affected by chemical reagents. 3. Fine *scrapings* of the kidney were agitated occasionally for two or three days in a test-tube, the water having been frequently changed. By this method the epithelial cells within the capsule were washed out, so that the space thus left between the tuft and the capsule became filled with water, which had soaked through the capsule; various points of the surface of the tuft could now be seen, covered by nucleated cells. Dr. Isaacs remarks:—"By the different processes just mentioned, I consider the existence of nucleated cells upon the surface of the Malpighian tuft, and consequently its analogy with other secreting organs, as conclusively demonstrated."

The author next considers the functions of the Malpighian body, which we shall notice more particularly when we come to the second paper, which treats especially of this subject. According to Mr. Bowman, whose opinions represent those of a majority of the profession at present, the Malpighian tuft lies entirely naked in its capsule, and its office is simply that of separating water from the blood, while the urea, lithic acic, and salts are separated by the epithelial cells of the tubes, from the blood of the venous plexus which surrounds them. The author differs from Mr. Bowman in his interpretation of the structure of the kidney of the boa constrictor, and in its supposed analogy with that of the human kidney; but the arguments and facts which he adduces would require more space than we can at present devote to the subject. We therefore pass on to that part which treats of the fibrous matrix of the kidney.

This was first described by Mr. Godolphin, in 1842. Notwithstanding its physiological and pathological importance, yet its very existence has been questioned, and is even now denied by several high authorities. It seemed, therefore, to the author, that the subject demanded fresh investigation. Prepared according to the process which he recommends—"a thin section of the kidney exhibits under the microscope a kind of mesh, network, or honey-comb arrangement, the cells of the honey-comb, however, having no bottom. In the natural condition of the kidney the smaller cells or openings transmit the tubes, the larger cells or openings are occupied by the Malpighian bodies," etc. etc. Plates of the fibrous matrix of the rat, dog, gray rabbit, raccoon, gray squirrel, hog, sheep, ox, horse, elk, moose, and human subject are here given.

Great pains were taken to prepare the matrix from perfectly healthy human kidneys, and Dr. Isaacs remarks that it is rare to find a human kidney *perfectly* healthy. "Out of more than five hundred subjects which I have examined in this city, I have seen but very few in which the kidney could be considered as in an entirely healthy condition." A view is given of the healthy fibrous matrix as contrasted with that taken from the kidney of an intemperate person. The matrix in the latter is shown to be withered and indurated. Many of the Malpighian bodies and tubes were constricted and diminished to less than half their natural size. A plate is given of the healthy matrix of the sheep, wherein it is shown that the minute vessels pass through the substance of the fibrous rings. Consequently when the matrix becomes indurated in the small "cirrhosed" kidney, this contraction by pressure on the vessels must interfere with the circulation, nutrition, and secretion of the organ, and their various morbid products, as pus, albumen, tube-casts, etc. may be found in the urine. In examining into the nature of the matrix, it was subjected to the action of various chemical reagents, by which it was concluded that the matrix is composed entirely of the white fibrous tissue, without any admixture of the colloid elastic tissue.

The proper substance of the kidney was found to consist—1, of the uriniferous tubes; 2, of the arteries, veins, and intermediate capillary plexus; 3, of the lymphatics; 4, the nerves; 5, of the plasma of the blood; 6, a few rounded and granular nuclei can sometimes be seen scattered in the substance of the matrix.

We now come to the second paper, which is upon the function of the Malpighian bodies.

In opposition to the views of Mr. Bowman, previously quoted, Dr. Isaacs believes that these bodies are actively engaged in secreting several of the proximate elements of the urine. This view was deduced from the following facts:—

1. "Urea, lithic acid, and the salts of the urine have been found to exist in the blood of healthy animals. In various diseases, and especially after extirpation of the kidneys, these substances are found in large proportion in the blood. It is then proved that they pre-exist in the blood, and are merely separated from it in the kidney.

2. "The renal artery *must* necessarily contain these proximate elements of urine, and as the Malpighian tufts are placed upon the terminal twigs of this artery, they *must* consequently enter the tuft.

3. "Most diuretics enter the circulation, and can be detected in the urine. They can arrive at the kidney only through the renal artery, and consequently must enter the Malpighian tuft."

4. It has been demonstrated in this paper that the Malpighian tuft is covered by nucleated cells, and is, therefore, a glandular structure, every way adapted for separating the proximate elements of urine.

5. "In serpents, alligators, turtles, etc., the Malpighian bodies abound throughout the cortical substance, and in some of these animals, throughout the whole structure of the kidney, yet their urine is secreted in the semi-solid state. It is not easy to reconcile this fact with the theory that the Malpighian body merely separates water from the blood.

6. "In the kidney of a patient who had been jaundiced for several years, I ascertained the presence of the coloring matter of the bile in the capsule of the Malpighian tuft, and in the commencement of the uriniferous tube. By the application of a drop of nitric acid the Malpighian bodies exhibited the usual changes of color which are seen when this acid is brought in contact with the bile. I therefore concluded that in this case the coloring matter had been separated by the Malpighian tuft, passed into the capsule, and downward into the uriniferous tube."

By tying the ductus communis, or common bile duct, in dogs, bile was also detected *in the Malpighian bodies*, and to some extent in the uriniferous tubes.

7. Many experiments were performed upon animals, of introducing into the stomach and intestines various coloring matters, which entered the circulation and were afterward detected in the kidney. Among these substances were finely pulverized indigo, Prussian blue, solutions of madder, Brazil-wood, cochineal, carmine, log-wood, Persian berries, animal charcoal, and lampblack. "In some Malpighian bodies, the various coloring substances were sometimes seen in the centre of the capsule, while a clear interval existed between the colored portion and the inner surface of the capsule; thus showing that the coloring matter had not left the tuft. In other instances, the central portion, corresponding to the tuft, was only slightly colored, while a deeply colored part was situated between the tuft and the internal surface of the capsule. *I think these experiments conclusively show, that the Malpighian tuft can and does separate coloring matters from the blood, which have been absorbed from the stomach and intestines, and have thus entered the circulation.*"

8. In two instances lithic acid was detected in the Malpighian bodies of the kidneys, which had been removed from the bodies of persons who had passed, before death, large quantities of the lithate of ammonia, and lithic acid crystals.

9. Some experiments were also made upon animals, to ascertain the effect of highly stimulating diuretics upon the secreting structures of the kidney. With this view, the nitrate of potassa, tincture of cantharides, turpentine, and phosphorus were severally given, at different times, to cats and dogs, and in all instances it was noticed that the Malpighian bodies were much congested, sometimes ruptured, and occasionally there was much congestion of the intertubular plexus.

10. With a view of causing a large amount of blood to be thrown upon the kidney, the abdominal aorta was ligatured in animals, a short distance above its bifurcation; the effect was to produce enormous distension and congestion of the Malpighian bodies and venous plexus. In connection with this, it is interesting to refer to the operation of Sir A. Cooper, for ligature of the abdominal aorta, in which case there was suppression of urine for more than twenty-four hours.

Incidentally it may be here mentioned, that the injected state of the vessels resulting from this operation greatly facilitates the investigation of the structure of the kidney. It was observed by the author, that the kidneys of persons who died from suppression of urine were often in a congested condition. This, taken in connection with the foregoing facts, would point to the importance of local depletion and counter-irritation in cases of suppression of urine and retention of urea in the blood, to the necessity of avoiding diuretics, or at least using them with great caution.

11. We may now speak of the conclusions to which the author has arrived, and what are the facts and deductions, which he thinks may be regarded as new or of an original character, or as tending to confirm or disprove the views of the best authorities on the subject.

*a.* The employment of certain new processes for displaying the structure of the kidney.

*b.* Contrary to the opinions of some of the highest authorities, the epithelium lining the urine tubes has been shown to be of the tessellated variety.

*c.* Ciliary motion, although weak and imperfect, has been seen in the kidneys of the mammalia.

*d.* The opinion of Mr. Bowman, relative to the connection of the Malpighian tuft with the capsule of the uriniferous tube, has been found to be indubitably correct.

*e.* Oval, nucleated cells exist upon the Malpighian tuft of the higher animals.

*f.* "*All the facts adduced in this paper lead to the conclusion that it is to the Malpighian body that we are to attribute the principal agency in the secretion of urine.*"

*g.* For the above and other reasons given in the paper, the commonly received physiological views of Mr. Bowman are not adopted.

*h.* A mode of readily exhibiting the fibrous matrix of the kidney, and views of this structure in different animals, have here been given.

*i.* The arrangement of the minute vessels as they pass through the substance of the matrix, and the effect of its construction upon the vessels, in cases of induration of the kidney.

*k.* The chemical and histological nature of the matrix.

*l.* Incidentally, some interesting facts have been stated relative to the absorption of substances which are generally considered to be insoluble, but which have been detected in the kidney, and also in other organs.

*m.* In conclusion, the author remarks that, "entering upon the examination of this subject without any pre-conceived theory, it has occupied much of my attention for some years past; and for the last twelve months I have labored upon the subject almost daily, sparing neither time, trouble, nor expense, and carefully guarding against arriving at any conclusion, until after long-continued and repeated examinations.

"The facts from which deductions have been drawn have all been obtained by the careful examination of the kidney in many animals, and especially in the frog, turtle, snake, alligator, fish, bird, mouse, rat, squirrel, cat, dog, raccoon, rabbit, hog, sheep, deer, elk, moose, ox, horse, black bear, rhinoceros, monkey, and in man." With regard to the plates, they were all drawn from nature with the exception of one only, the greatest care having been taken to insure accuracy in every instance.

*Experimental Researches Relative to the Nutritive Value and Physiological Effects of Albumen, Starch, and Gum, when singly and exclusively used as Food; being one of the Prize Essays of the American Medical Association for* 1857. By WM. HAMMOND, M.D., Assistant Surgeon, U.S.A.

The essay before us is a remarkable addition to our knowledge of the physiology of nutrition, and the metamorphosis of tissue. It is not less remarkable as a straight forward autobiographical record of the sacrifice of comfort in the pursuit of a scientific result. We can easily give the main results of Dr. Hammond's researches, but nothing except a complete analysis of the whole paper would enable us to render justice to the industry and self-devotion of the author. He who is at once the observer and the observed, in such an investigation, has need of a degree of energy and endurance with which few men are endowed. So far, however, science has made no demand to which her votaries have not responded so eagerly that men are found ready to submit to any hardships, endure any mode of life, to gain the simple prize which she sets before them.

To show how the essay before us is a sermon on this text, we will analyze it rapidly and briefly. Eighteen pages are taken up with general remarks, statement of chemical methods employed, and a determination thereby of Dr. Hammond's standard of excretion, weight, etc. The researches determining these latter points extend over five days. They are merely the necessary preparation, and we learn from them, for future comparison, the following particulars:—1st. The average daily excretion of water, urea, uric acid, chlorine, sulphuric acid, phosphoric acid, etc., in the form of urine. 2d. The weight of the fæces, their amount of contained water, and the proportions of their ether, alcoholic and aqueous extracts, and insoluble residue. 3d. The weight of the body, the pulse, and the temperature of the body; and 4th. The normal proportions of the various ingredients of the observer's blood. We are also told all essential particulars as to the height, size, habits, and history of the subject of these experiments, so that, on the whole, Dr. Hammond must be about as well and accurately described as any other specimen of the genus.

With this preparation Dr. Hammond began his experiments. During ten days he eat no food but albumen from bullock-blood, and drank only distilled water, or snow water. All other conditions remained unchanged. Every day the ingesta and egesta were carefully weighed, and the pulse, temperature, and feelings noted. It is a curious record. On the sixth day the albumen became nauseating, and on the seventh day *albumen appeared in the urine.* On the tenth day Dr. Hammond became very weak, slept ill, and experienced a severe diarrhœa and some mental confusion. He recovered his usual health a few days after the close of the experiment, upon resuming his usual diet. On comparison of his general condition on the tenth day, with the standard arrived at before he began to take the albumen, Dr. Hammond came to certain conclusions, well supported by tables of his analyses, which we are sorry not to have space to quote. Under this exclusively albuminous diet, the urine decreased absolutely, both as to solid and fluid ingredients. Relatively, however, the solids were increased in amount. Thus the urea increased, but not largely. The uric acid increased considerably. Chlorine, sulphuric, and phosphoric acids were lessened.

The whole amount of fæces increased, owing to the diarrhœa on the last two days; otherwise the mean daily amount of fæces was less than in health. The fæcal solids, as well as the ether and aqueous extracts of the fæces, were diminished. The water of the stools and the alcohol extracts were increased.

The weight of the body fell off. The pulse rose. The temperature fell. Meanwhile the blood lost water, blood corpuscles, soluble inorganic salts, and fat, gaining, on the contrary, solids, fibrin, albumen, and extractive matter. From these data Dr. Hammond proceeds to discuss a number of physiological problems, on which we cannot now dwell.

Article second of the essay relates a similar series of experiments upon the ingestion of starch as the sole aliment. The results are of equal interest. At the close of ten days of this exclusively farinaceous diet, Dr. Hammond was in a condition of ill health, from which he recovered but slowly. From the sixth day his urine exhibited reaction indicative of the presence of sugar. The pulse rose, the temperature increased, headache and indigestions came on, and the system assumed an inflammatory tendency, which showed itself in fever, etc., and in the difficulty with which even the slightest scratches healed. Finally a comparison of the results thus obtained with those of the standard series evolved the following conclusions:—

The urine fell off in amount, and all its constituents were lessened excepting those classified by Dr. H. as "residue," a term which must include coloring and extractive matter, rich in carbon, creatine, sugar, etc. This loss of urinary solids is explained by Dr. H. as due to the fact that "these substances must have been entirely derived from the disintegrated tissues of the body; the food containing no material from which they could have been elaborated. The amount of fæces is stated to have been lessened, as well as that of each of its constituents. Curiously enough the weight of the body declined but 389·71 grains; proof sufficient that a good deal of the starch was in some form assimilated, most probably, our author thinks, as fat derived from the metamorphosis of the amylaceous food so exclusively employed. On the close of these experiments the blood, which was again examined, showed an increase of fat, fibrin, and extractive matter—results easily to be anticipated, after the use of a starchy diet.

Article third describes Dr. Hammond's observations upon the exclusive use of gum. It is unnecessary to analyze freely this portion of the essay. Four days of a diet of gum Arabic produced so much physical indisposition, and such an invincible desire for other food, that even the self-devotion of Dr. Hammond gave way to the instincts of the system, and he wisely contented himself with the results thus far acquired, after four days of gum diet. He found, at this time, the urine increased as to its fluid only, the solids having fallen off. The fæces increased very largely in amount, owing to the great quantities of undigested gum which they contained, scarcely any of this substance seeming to have been absorbed. The loss of weight was very considerable, and the author, properly, we think, concludes, as did Lehman, that this article is useless, as a diet, in small amount, and hurtful, he adds, in larger amount.

The physiological inferences which naturally follow upon these researches are of extreme interest, and will repay very amply a careful reading.

---

*On the Constitution and Physiology of the Bile.* By JNO. C. DALTON, M.D., etc. Am. Jour. Med. Sci., Oct. 1857.

THIS is a very interesting essay, chiefly devoted to the chemistry of the bile. Dr. Dalton has repeated and confirmed the researches of Strecker, etc., and has also added, we believe, some original results of his own. If, however, in every paper supposed, like this one, to be novel in its views or experimental means, the author would distinctly state at the close what he claimed as new and original, we should more distinctly comprehend how much science had gained by him. Those who are not versed in organic chemistry, and who as physicians read such articles as this one, as matters of general medical interest, are too apt to attribute to the author of the paper a larger share of what appear as its results than he is of right entitled to. As we are conscious that such an intention is quite remote from Dr. Dalton's wishes and aims, we may here state that the evil thus alluded to is one which has been common to many physiological and other papers upon this side of the ocean, and is, we think, so easy of remedy, and so obvious, that we feel bound to call attention to it whenever it occurs in the essays before us.

At the close of his chemical review, Dr. Dalton examines the question of the time after feeding when bile is most abundantly thrown into the intestines. He correctly thinks that most of the former experiments by *biliary* fistula merely indicate the time of greatest formation of bile by the liver. His own plan, by making a permanent *duodenal* fistula, gives the chance of determining the period of greatest supply to the gut. He thus learned that the supply immediately after feeding is greatest within the first hour; after which it remains constant, or nearly so, as to absolute amount; although, of course, as the supply of food from the stomach and digestive process decreases, the relative proportion of bile to duodenal contents rises. From the eighteenth to the twenty-fourth hour after feeding, there is, however, a marked diminution in the supply.

In regard to its digestive uses, Dr. Dalton determines anew that it precipitatés the albuminous matters from gastric juice; that it is the biliary substances, apart from the coloring matter, which cause this precipitate, the pigments of the bile being non-essential to its production. Our author also casts some doubt upon Professor Bernard's view of the physiology of intestinal digestion.*

Finally, Dr. Dalton ingeniously investigates the question of what becomes of the bile. It thus appears, that Pettenkofer's test fails to indicate the existence of bile in the large intestines of the dog, while in the small guts it is constantly found. We may add that Dr. Dalton also sought for the bile-matters in the portal blood, but failed to detect them, notwithstanding the probability of their absorption.

---

* See review of V. Bernard's opinions, in the July number of this journal.

There are many minor points which we cannot note, but which give added interest to the main subjects. Dr. Dalton's statements of the means used in his investigation are clearly made, and the crystalline forms of the bile-salts are prettily illustrated by wood-cuts.

---

*The Excito-secretory System of Nerves: Its relations to Physiology and Pathology; being one of the Prize Essays of the Am. Med. Association for* 1857. By H. F. CAMPBELL, M.D., Professor of Special and Comp. Anat. in the Med. Coll. of Georgia, etc. etc., May, 1857.

"The object of this present essay," says the author, "is to develope more fully a function of the nervous system, which, though enunciated and described nearly seven years ago, in this country, is only now beginning to be fully recognized by the profession in Europe. We refer to that function which has been called the excito-secretory, and which results from the relations subsisting between the excitor or sensitive nerves of the cerebro-spinal, and the secretory branches of the ganglionic system."

With this preface—the meaning of the term excito-secretory being plain enough—we have only to state that Dr. Campbell sees, in the sympathetic, a nervous system through which the processes of secretion and nutrition may be variously affected. As above stated, our author puts forward a claim to having first set forth this doctrine in 1850; (*Southern Med. and Surg. Jour.*, vol. vi., p. 321, 1850.) Dr. Marshall Hall, who seems also to have been struck with the same idea, finally admitted the priority of claim of the Georgia professor; (*London Lancet*, March, 1857; *Lond. Med. Gaz.*, June and July, 1857; also, *Tr. Am. Med. Assoc.*, vol. vi., 1853.) We find also, in Brown-Séquard's last essay, a note on the same matter. He admits Dr. Campbell's claim as prior to Dr. Hall's, but thinks that both of these gentlemen were not aware of how much had been done in Germany toward the subject of reflex secretion. He also advances a claim of his own, dating back to 1849, and mentions other authors in the same connection.

Now, there is no doubt that very many persons had explained individual facts by a reference to reflex influence, but we do not know that any one has hitherto generalized as broadly in this matter as our author has, or that any one has before this singled out and enunciated the special value of the two nervous systems concerned, as he supposes, in the phenomena commented upon. Assuredly, the general idea, as presented by Dr. Campbell, will come before most medical minds as on the whole a remarkable novelty; although, perhaps, each one of us may have applied the term and meaning reflex, to some single fact, or even to some partial collection of facts connected with secretion.

The first half of the essay is an endeavor to show that the sympathetic is a nerve of secretion and nutrition, distributed so as best to fulfil these functions. We note here, as very clever and well done, Dr. Campbell's commentary on Magendie and Alcock, in relation to the various experiments upon the fifth pair.

The second half of the essay deals with pathological facts, which are applicable under Dr. Campbell's views. The author has, however, by no means exhausted the examples, either normal or abnormal, which may be used as illustrations of his theory. Since he has given us no new facts of his own creation, we have refrained from quoting where nothing but a thorough and careful reading of the whole statement would be of any use. Dr. Campbell adopts at the outset Cousin's phrase,—"That observation becomes experiment when used in severe processes of induction," and accordingly adds no novel experimental elucidations. This exclusive use of the facts prepared by others, perhaps insures an impartial view of those facts; but, it may also be added as a mere result of experience, that inductive reason has never been so felicitous as when hand in hand with experimental skill. We hope still to see Dr. Campbell's views more fully developed, and treated yet further by the keen criticism of multiplied experiment.

---

*Life: Its Relations, Animal and Mental.* By J. D. BRUNS, A.M., M.D.

This is a pamphlet of fifty-eight pages, by the accomplished physician who has just taken charge of the *Charleston Medical Journal and Review*, in place of Dr. Happoldt, the former editor. The little brochure before us is a remarkably intelligent sketch of the various theories, old and new, connected with mental physiology, and the relations of mind and body, while, at the same time, it is also an agreeable and often original commentary upon their value. In many places, indeed, the writer is eloquent and forcible, almost everywhere expressing himself with a clearness of definition not usually met with in more ambitious works. Many of Dr. Bruns' criticisms upon Professor Carpenter's views are such as we entirely agree with, and we wonder with Dr. B. how any mind can regard as other than intelligent the feats, etc. of some of the animals, and even insects, to whom the English physiologist is only willing to allow the gift of instinct. The argument against this view is excellently stated by our author, and, like the rest of his pamphlet, is pleasant and easy reading. Certain other parts we had marked for dispute, but the abstract points of metaphysics are not to be settled in a few sentences, and we are disinclined to contradict without also endeavoring to make good our assertions,—a task for which time and space are equally lacking.

---

*Essay on the Physiology of the Nervous System, with an Appendix on Hydrophobia.* By B. HASKELL, M.D., Rockport, Mass.; 8vo, pp. 88.

*Essay on Muscular Motion.* By J. H. WATTERS, M.D., Professor of Phys. in the St. Louis Med. Col.

There is but little danger nowadays of any theory being allowed to enjoy a long reign of quiet authority, and the prevailing hypotheses which seek to explain the functions of nerves, etc. are not likely to prove exceptions, or to escape attack. In fact, every few months bring us pamphlets like those before us, which, although they offer no new facts, serve at least to point out the weaknesses of accepted dogmas. Dr. Haskell's essay is an ingenious criticism of prevailing views, and a protest against the materialism to which he thinks they must inevitably lead us. Unfortunately we have not the space to reply in full to the author's arguments, which would require as much space for their demolition as he has occupied for their construction. The new theory with which Dr. Haskell proposes to replace the present system of explaining the facts of the nervous functions is, however, but a hypothesis, and not altogether novel.

Dr. Waters' essay is one of a series. It is almost altogether an attack upon Professor Carpenter's views, and does not profess to offer any satisfactory substitute for the faith it is intended to destroy.

